# Supplementary material for: Interaction between central obesity and frailty on the clinical outcome of peritoneal dialysis patients
Source: PLoS One. 2020 Oct 26;15(10):e0241242. doi: 10.1371/journal.pone.0241242 (PMC7588087; doi:10.1371/journal.pone.0241242)
Supplement: S1 Checklist — (DOCX) [file pone.0241242.s001.docx]

**Modified STROBE Statement—checklist of items that should be included in reports of observational studies (Cohort/Cross-sectional and case-control studies)**

|  | Item No | Recommendation |
| --- | --- | --- |
| **Title and abstract** | 1 | (*a*) Indicate the study’s design with a commonly used term in the title or the abstract  The study design i.e. prospective observation cohort study was stated in Abstract part. |
|  |  | (*b*) Provide in the abstract an informative and balanced summary of what was done and what was found  Summary was stated in Methods, Results and Conclusion parts in Abstract |
| Introduction | | |
| Background/rationale | 2 | Explain the scientific background and rationale for the investigation being reported  The rationale including findings from current literature was mentioned |
| Objectives | 3 | State specific objectives, including any prespecified hypotheses  The objectives – To examine the interaction between frailty and obesity on the clinical outcome of PD patients were mentioned in Abstract part. |
| Methods | | |
| Study design | 4 | Present key elements of study design early in the paper  The study design I.e. prospective observational cohort study was stated. |
| Setting | 5 | Describe the setting, locations, and relevant dates, including periods of recruitment, exposure, follow-up, and data collection  These were mentioned in the subsections of Study Population, Assessment of Frailty, Anthropometric and body composition assessment, Pulse Wave Velocity and Outcome measures within the Patients and Methods part. |
| Participants | 6 | (*a*) *Cohort study*—Give the eligibility criteria, and the sources and methods of selection of participants. Describe methods of follow-up  *Case-control study*—Give the eligibility criteria, and the sources and methods of case ascertainment and control selection. Give the rationale for the choice of cases and controls  *Cross-sectional study*—Give the eligibility criteria, and the sources and methods of selection of participants  Eligible and exclusion criteria were mentioned in the Study population subsection in Patients and Methods part. |
| Variables | 7 | Clearly define all outcomes, exposures, predictors, potential confounders, and effect modifiers. Give diagnostic criteria, if applicable  The diagnostic criteria of frailty, central and general obesity, as well as definition of outcomes were described in detail in Patients and Methods section. |
| Data sources/ measurement | 8* | For each variable of interest, give sources of data and details of methods of assessment (measurement).  The diagnostic criteria of frailty, central and general obesity were described in detail in Patients and Methods. |
| Bias | 9 | Describe any efforts to address potential sources of bias  The potential bias was addressed by appropriate statistical tests. Such issue was further discussed in the Discussion part. |
| Study size | 10 | Explain how the study size was arrived at (if applicable)  Not applicable. |
| Quantitative variables | 11 | Explain how quantitative variables were handled in the analyses. If applicable, describe which groupings were chosen and why  The interpretation of frailty scores, central and general obesity were explained. |
| Statistical methods | 12 | (*a*) Describe all statistical methods, including those used to control for confounding  The statistical methods were explained in detail in the Statistical Analysis section. |
|  |  | (*b*) Describe any methods used to examine subgroups and interactions  The statistical methods were explained in detail in the Statistical Analysis section. |
|  |  | (*c*) Explain how missing data were addressed  There was no missing data in our series. |
|  |  | (*d*) *Cohort study*—If applicable, explain how loss to follow-up was addressed  *Case-control study*—If applicable, explain how matching of cases and controls was addressed  *Cross-sectional study*—If applicable, describe analytical methods taking account of sampling strategy  The definition of censoring events was described in Outcome measures part. |
|  |  | (*e*) Describe any sensitivity analyses  It is not applicable. |
| Results | | |
| Participants | 13* | (a) Report numbers of individuals at each stage of study—eg numbers potentially eligible, examined for eligibility, confirmed eligible, included in the study, completing follow-up, and analyzed  The number of subjects i.e. 267 subjects were stated. |
|  |  | (c) **Use of a flow diagram**  Figure 1 is the flow diagram |
| Descriptive data | 14* | (a) Give characteristics of study participants (eg demographic, clinical, social) and information on exposures and potential confounders  The subjects’ characteristics were summarised in Table 1 and Supplementary Table 1 |
|  |  | (b) Indicate number of participants with missing data for each variable of interest  There was no missing data. |
|  |  | (c) *Cohort study*—Summarise follow-up time (eg, average and total amount)  Follow-up time (2 year) was stated |
| Outcome data | 15* | *Cohort study*—Report numbers of outcome events or summary measures over time  Outcome events were stated in Results section. |
|  |  | *Case-control study—*Report numbers in each exposure category, or summary measures of exposure |
|  |  | *Cross-sectional study—*Report numbers of outcome events or summary measures |
| Main results | 16 | (*a*) Give unadjusted estimates and, if applicable, confounder-adjusted estimates and their precision (eg, 95% confidence interval). Make clear which confounders were adjusted for and why they were included  The results were stated in the Results part and were summarised in Tables 1 to 6 and Supplementary Tables 1-2. The list of confounders adjusted, and reasons why they were included were explained in Statistical analysis part. |
| Other analyses | 17 | Report other analyses done—eg analyses of subgroups and interactions, and sensitivity analyses  Not applicable. |
| Discussion | | |
| Key results | 18 | Summarise key results with reference to study objectives  The key results were presented in the first paragraph in Discussion part. |
| Limitations | 19 | Discuss limitations of the study, taking into account sources of potential bias or imprecision. Discuss both direction and magnitude of any potential bias  The limitations of the study were discussed in the second last paragraph of Discussion part. |
| Interpretation | 20 | Give a cautious overall interpretation of results considering objectives, limitations, multiplicity of analyses, results from similar studies, and other relevant evidence  The overall interpretation of results with reference to other similar studies were discussed in Discussion part. |
| Generalisability | 21 | Discuss the generalisability (external validity) of the study results  The generalisability (external validity) was discussed in the Discussion part. |

*Give information separately for cases and controls in case-control studies and, if applicable, for exposed and unexposed groups in cohort and cross-sectional studies.

**Note:** An Explanation and Elaboration article discusses each checklist item and gives methodological background and published examples of transparent reporting. The STROBE checklist is best used in conjunction with this article (freely available on the Web sites of PLoS Medicine at http://www.plosmedicine.org/, Annals of Internal Medicine at http://www.annals.org/, and Epidemiology at http://www.epidem.com/). Information on the STROBE Initiative is available at www.strobe-statement.org.
