# Supplementary material for: Interaction between central obesity and frailty on the clinical outcome of peritoneal dialysis patients
Source: PLoS One. 2020 Oct 26;15(10):e0241242. doi: 10.1371/journal.pone.0241242 (PMC7588087; doi:10.1371/journal.pone.0241242)
Supplement: S1 Table — (DOCX) [file pone.0241242.s003.docx]

### S1 Table. Baseline clinical and demographic data, statistical tests results

|  | **Not frail** | **Frail** | **P value** |
| --- | --- | --- | --- |
| No. of patients | 147 | 120 |  |
| Age (year) | 61.1 ± 11.8 | 64.2 ± 12.2 | p = 0.03^a^ |
| Sex (M:F) | 75 : 72 | 56 : 64 | p = 0.5 |
| Duration of dialysis (months) | 44.3 ± 46.9 | 51.8 ± 57.4 | p = 0.24^a^ |
| Blood pressure (mmHg)    Systolic    Diastolic | 142.8 ± 18.9  75.8 ± 12.4 | 142.6 ± 20.9  73.4 ± 12.7 | p = 0.9^a^  p = 0.12^a^ |
| Renal Diagnosis, no of cases. (%)    Glomerulonephritis    Diabetic nephropathy    HTN    Polycystic kidney    Urological disease    Other / unknown | 51 (34.7%)  42 (28.6%)  18 (12.2%)  5 (3.4%)  9 (6.1%)  22 (15%) | 33 (27.5%)  56 (46.7%)  12 (10%)  3 (2.5%)  2 (1.7%)  14 (11.6%) | p = 0.081^b^ |
| Comorbid disease, no of cases (%)    DM    IHD    CVA | 55 (37.4%)  19 (12.9%)  16 (10.9%) | 66 (55.0%)  19 (15.8%)  31 (25.8%) | p = 0.004^b^  p = 0.5^b^  p = 0.001^b^ |
| Charlson Comorbidity Index | 5.1 ± 2.2 | 5.6 ± 2.2 | p = 0.045^a^ |
| Nutritional Status    MIS    SGA | 6.57 ± 3.13  5.38 ± 0.83 | 9.00 ± 3.46  4.84 ± 0.92 | p < 0.001^a^  p < 0.001^a^ |
| Total weekly Kt/V | 1.88 ± 0.49 | 1.76 ± 0.40 | p = 0.051^a^ |
| Residual GFR (ml/min/1.73m2) | 1.70 ± 2.02 | 1.04 ± 1.74 | p = 0.01^a^ |
| NPNA (g/kg/day) | 1.07 ± 0.25 | 1.03 ± 0.30 | p = 0.23^a^ |
| Biochemical parameters  Hemoglobin (g/dL)  Albumin (g/L) | 9.72 ± 1.31  34.10 ± 4.15 | 9.25 ± 1.26  33.46 ± 4.16 | p = 0.009^a^  p = 0.3^a^ |
| Pulse Wave Velocity (m/sec)    CF-PWV    CR-PWV | 10.93 ± 2.38  10.34 ± 1.75 | 11.19 ± 2.40  10.48 ± 1.80 | p = 0.4^a^  p = 0.5^a^ |
| Peritoneal Transport State-  4-hour dialysate/plasma creatinine | 0.64 ± 0.13 | 0.65 ± 0.12 | p = 0.562^a^ |
| Helper-assisted PD, no of cases (%)  Type of peritoneal dialysis, no of cases (%)  CAPD  CCPD  NIPD  Icodextrin dialysate use, no of cases (%) | 8 (5.4%)  127 (86.4%)  4 (2.7%)  16 (10.9%)  39 (26.5%) | 24 (20.0%)  106 (88.3%)  2 (1.7%)  12 (10.0%)  34 (28.3%) | p < 0.001^b^  p = 0.817^b^  p = 0.742^b^ |
| Concomitant medications, no of cases (%)  Aspirin  Beta blocker  RAS blocking agent  Calcium channel blocker  Statin  Calcium containing phosphate binder  Vitamin D supplement  Erythropoiesis-stimulating agents | 35 (23.8%)  99 (67.3%)  79 (53.7%)  125 (85.0%)  64 (43.5%)  115 (78.2%)  64 (43.5%)  112 (76.2%) | 42 (35.0%)  72 (60.0%)  77 (64.2%)  90 (75.0%)  62 (51.7%)  84 (70.0%)  62 (51.7%)  82 (68.3%) | p = 0.045^b^  p = 0.213^b^  p = 0.086^b^  p = 0.039^b^  p = 0.186^b^  p = 0.125^b^  p = 0.186^b^  p = 0.152^b^ |

Data are expressed as mean ± standard deviation, and are compared by ^a^Student's t-test and ^b^Chi-square test.

HTN, hypertensive nephrosclerosis; DM, diabetes mellitus; IHD, ischemic heart disease; CVA, cerebrovascular accident; MIS, Malnutrition Inflammation Score; SGA, Subjective Global Assessment; GFR, glomerular filtration rate; NPNA, normalized protein nitrogen appearance; CF-PWV, carotid-femoral pulse wave velocity; CR-PWV, carotid-radial pulse wave velocity; PD, peritoneal dialysis; CAPD, continuous ambulatory peritoneal dialysis; CCPD, continuous cycler peritoneal dialysis; NIPD, nocturnal intermittent peritoneal dialysis; RAS blocking agent, renin angiotensin system-blocking agent.
