# Supplementary material for: Interaction between central obesity and frailty on the clinical outcome of peritoneal dialysis patients
Source: PLoS One. 2020 Oct 26;15(10):e0241242. doi: 10.1371/journal.pone.0241242 (PMC7588087; doi:10.1371/journal.pone.0241242)
Supplement: S2 Table — (DOCX) [file pone.0241242.s004.docx]

### S2 Table. Relation between hospitalization and general versus central obesity in frail patients.

|  | **No central obesity** | **Central obesity** | **P value*** | **No general obesity** | **General obesity** | **P value*** |
| --- | --- | --- | --- | --- | --- | --- |
| Number of patients | 72 | 48 |  | 104 | 16 |  |
| Number of hospitalization |  |  |  |  |  |  |
| All-cause | 4.48 ± 4.39 | 4.29 ± 4.22 | p = 0.872 | 3.98 ± 3.79 | 6.88 ± 6.18 | p = 0.085 |
| Cardiovascular event | 0.54 ± 1.07 | 0.38 ± 0.81 | p = 0.527 | 0.43 ± 0.94 | 0.50 ± 0.82 | p = 0.587 |
| Duration of hospitalization (days) |  |  |  |  |  |  |
| All-cause | 55.88 ± 76.46 | 47.89 ± 63.89 | p = 0.824 | 46.45 ± 64.27 | 81.19 ± 91.03 | p = 0.063 |
| Cardiovascular event | 10.88 ± 30.30 | 4.06 ± 9.99 | p = 0.393 | 7.08 ± 22.12 | 4.88 ± 8.49 | p = 0.728 |

*Data are compared by Mann Whitney U test.
