# Supplementary material for: Interaction between central obesity and frailty on the clinical outcome of peritoneal dialysis patients
Source: PLoS One. 2020 Oct 26;15(10):e0241242. doi: 10.1371/journal.pone.0241242 (PMC7588087; doi:10.1371/journal.pone.0241242)
Supplement: Attachment — Submitted filename: Response to Reviewers.docx [file pone.0241242.s006.docx]

2 October 2020

Editor in Chief

*PLoS One*

Dear Editor in Chief,

Re: ‘Interaction Between Central Obesity and Frailty on the Clinical Outcome of Peritoneal Dialysis Patients’ (PONE-D-20-19119)

Thanks for your letter on 21 September 2020. The following revisions have been made according to your recommendations:

Reply to Editor and Reviewer 2

1. **Can you come with a more crisp and clear clinical message and comment on how much weight can be given to a study in small numbers of patients when outcome differences are barely or not significant.**
   Page 20, paragraph 2, line 16-22: We acknowledged the small number of patients that we recruited in our study is one of our limitations. It is also one of the reasons that may lead to barely significant or insignificant outcome differences. Despite so, our results, which represent the first published data in this aspect, demonstrated the trend of outcome and provided valuable information by suggesting a potential association and interaction with the adverse outcomes. We suggest future research with a larger sample size to look into any association with the outcomes that we investigated. This limitation and recommendation are now included in the Discussion Part.
2. **There is no explanation why higher Waist:Hip ratio predicts CV events in general population but is supposedly protective in non-frail PD population.**
   Page 19, paragraph 1, line 16-23: Abdominal obesity is also reported to be a protective factor against mortality in patients with stable coronary artery disease patients, and heart failure patients especially those with reduced ejection fraction. The proposed mechanisms are related to the extra energy reserve provided by visceral fat, and the anti-inflammatory and endotoxin-neutralizing effects mediated by a raised lipoprotein level. In addition, central obese individuals also seek medical attention at the earlier stage of acute illnesses as they tend to be more symptomatic, and their renin-angiotensin-aldosterone system is more attenuated, causing a higher blood pressure and better tolerance to cardioprotective medications. These findings and proposed mechanism are now included in the Discussion Part.

We would like to resubmit the manuscript, with the changes highlighted in yellow, for consideration of publication in the PLoS One. Thank you for reviewing our article and we look forward to hearing your favorable reply.

Yours sincerely,

Dr. Gordon CK Chan

for Drs. JKC Ng, KM Chow, BCH Kwan, VWK Kwong, WF Pang, PMS Cheng, MC Law, CB Leung, PKT Li and CC Szeto
